# Non-small-cell lung cancer in a French department, (1982–1997): management and outcome

**DOI:** 10.1038/sj.bjc.6602342

**Published:** 2005-01-25

**Authors:** J Foeglé, G Hédelin, M P Lebitasy, A Purohit, M Velten, E Quoix

**Affiliations:** 1 Laboratoire d'épidémiologie et de santé publique, Université Louis Pasteur, Strasbourg, France; 2 Service de Pneumologie Lyautey, Hôpitaux Universitaires, 1, Place de l'Hôpital, 67091 Strasbourg, Cedex, France

**Keywords:** non-small-cell lung cancer, management, survival, surgery, radiotherapy, chemotherapy

## Abstract

Addition of chemotherapy to the treatment of non-small-cell lung cancer (NSCLC) resulted in a modest but clear improvement in the survival of selected patients. To ascertain if this translates to improved survival in the whole population of patients, we conducted a retrospective population-based study of a sample of 1738 patients diagnosed with primary NSCLC in a French department between 1982 and 1997. The proportion of women, metastatic cases and adenocarcinoma changed significantly over time, as did their management: use of chemotherapy alone increased from 9.7 to 28.1% (*P*<0.0001), while the use of radiotherapy alone decreased from 32.2 to 9.4% (*P*<0.0001). The 5-year survival probability was 15.7 % for all patients and 32.6% for those with resectable disease. The 1- and 2-year survival probabilities were 38.2 and 15.6% in locally advanced disease, and were, respectively, 16.8 and 5.2% in metastatic disease. Disease extent and histological subtype were significant independent prognostic factors. Survival of resectable disease was longer among patients treated with surgery or surgery plus chemotherapy, while better outcomes for locally advanced disease were associated with radiation plus chemotherapy. In metastastic disease, patients treated by classical agent without platin or palliative care only had the shortest survival. Despite changes in treatment in accordance with the state-of-the-art, overall survival did not improve over time. It is not unlikely that more patients with bad PS were diagnosed during the latter end of the study period. This could at least partially explain the absence of detection of an overall improvement in survival.

Lung cancer, one of the most common malignancies, is the leading cause of cancer death among men through out the industrialised world (Ferlay *et al*, 2001). Since 1987, lung cancer has caused more deaths than breast cancer among North American women (US Public Health Service, 2001), and it is now the third leading cause of cancer deaths among French women (Hill and Doyon, 2004).

Non-small-cell lung cancer accounts for approximately 75% of all lung cancers. Its prognosis is poor: 5-year survival rates range from 60% in resected stage IA disease to 5% in stage IIIB and 1% in stage IV (Mountain, 1997). At diagnosis, approximately 75% of patients have tumours classified as stage III or IV disease (Fry *et al*, 1999).

Management of Non-small-cell lung cancer (NSCLC) has changed substantially over the past two decades with an increasing use of chemotherapy. At the beginning of the 1980s, the standard treatments were surgery for resectable tumours, mediastinal irradiation for locally advanced disease and supportive care for metastatic disease. Meta-analyses (Souquet *et al*, 1993; NSCLCCG, 1995) of the clinical trials comparing chemotherapy *vs* no chemotherapy report modest but clear improvement in survival for patients with metastatic or locally advanced disease who are treated with cisplatin-based chemotherapy and a trend towards improved survival with adjuvant chemotherapy in resectable disease. (The favourable impact of adjuvant chemotherapy has just been demonstrated very recently; The IALT Collaborative Group, 2004). Preoperative chemotherapy strategies might improve survival in resectable stage IIIA disease (Roth *et al*, 1994) as well as in stages I and II (Depierre *et al*, 2002).

To evaluate the effect on survival of these management changes in a population-based study, we conducted a retrospective study over a 16-year period of a sample of 1738 patients diagnosed with NSCLC in the department of Bas-Rhin (northeastern France).

## PATIENTS AND METHODS

### Patients

The Bas-Rhin population-based cancer registry furnished us with a list of patients with confirmed NSCLC. This department had a population of roughly 1 000 000 inhabitants during the study period (INSEE, 1982, 1990, 1999). We included 40% of the 5071 cases diagnosed between 1982 and 1997 and randomly selected 2028 patients, stratified by year of diagnosis.

We reviewed the medical records of each patient and excluded those with *in situ* carcinoma or a combination of small-cell and NSCLC. The following data were collected: age, sex, date of diagnosis, histological subtype, investigations performed for diagnostic and staging purposes and disease extent. We did not attempt to assign stages retrospectively but used the stage assigned by the patient's physician at diagnosis: resectable disease, locally advanced unresectable disease and metastatic disease. The stage was classified as undetermined if the chart did not report the local extent of the disease at diagnosis and if no metastatic site was detected.

Initial treatment (surgery, radiotherapy, chemotherapy, palliative care) and any clinical trial participation were also recorded. Chemotherapy was divided into classical agents and more recent agents (vinorelbine, gemcitabine, irinotecan) and subdivided into protocol with or without platin.

Survival was estimated from the date of pathological diagnosis. The end of follow-up was 31 December 2002.

### Statistical methods

#### Descriptive analysis

We defined four consecutive periods covering 4 years each. Differences between proportions were assessed with either Pearson's *χ*^2^ test or Fisher's exact test. Differences between the means of continuous variables were evaluated with Student's *t*-test or, if the comparison involved more than two groups, one-way analysis of variance. The 8.2 SAS software package (SAS Institute, Cary, NC, USA) was used to analyse the data.

#### Survival analysis

Our study covered 16 years. To eliminate the effect of improved survival for the other pathologies and the effect of age-related mortality, we conducted simple and multiple regressive analysis of relative rather than crude survival (Estève *et al*, 1990) with the RELSURV software package (Hédelin, 2003). The variables with a *P*-value of 0.20 or less in the simple regressive analysis were entered in the multiple regressive analysis. Sex and period were forced in the model. The proportionality assumption has been verified using interaction with time. Interactions between variables were tested, and none was statistically significant.

#### Power considerations

Our principal objective was to determine whether relative survival globally changed in NSCLC patients between 1982 and 1997. Using registry data, we estimated that the 5-year relative survival rate was 15% in the early 1980s, and we wanted to be able to detect an increase of at least five points, that is, a 5-year relative survival rate of 20% at the end of the 1990s under the assumption of the proportionality of the risks. With the formula given in Jung and Hui (2002) and assuming a one-sided test with an alpha level of 0.05 and a power of 0.80, we estimated that a sample size of 490 patients would be required in each of the four periods.

## RESULTS

### Descriptive analysis

We excluded 290 of the 2028 patients randomly selected from the registry: 197 patients had missing medical records, and 93 did not meet the inclusion criteria. The study thus included 1738 patients, 89.8% of the 1935 eligible.

The 197 eligible patients excluded from the study differed in some ways from the 1738 patients included. The proportion of women in these groups was 17.2 and 9.2%, respectively (*P*=0.0001), the median age 69.0 and 63.0 years (*P*<0.0001), and their median survival time (MST) substantially different (21.6 *vs* 45.3 weeks, *P*=0.00006). The proportion of cases with missing data decreased significantly over the study period from 15.5 to 9.5% (*P*=0.0109). Table 1 summarises the patients' characteristics and their trends over time.

The proportion of metastatic disease increased over the study period, and the histological distribution changed, especially in the last period, adenocarcinoma became more frequent and squamous cell carcinoma less so (Table 1).

Table 2 reports the diagnostic and staging procedures performed according to the period.

On 31 December 2002, 5.9% of the patients were still alive, 92.8% had died and 1.3% had been lost to follow-up.

From period 1 through period 4, the mean number of metastatic sites detected increased from 0.36 to 0.57 (*P*=0.0057) and the mean number of extra-thoracic imaging procedures from 1.79 to 2.87 (*P*<0.0001). These increases were significantly correlated (*r*=0.13, *P*<0.0001).

In all, 17 cases discovered incidentally at autopsy or who died before diagnosis were excluded from the following analyses.

Treatments for each stage differed significantly from the first to the last study period (*P*<0.0001, Table 3). Data for cases involving bilateral disease and undetermined stage are not shown. The percentage of patients treated with mediastinal irradiation alone decreased over time from 32.2 to 9.4% (*P*<0.0001) for every stage. On the other hand, the combined use of chemo- and radiotherapy increased significantly over time for the locally advanced stage and the use of chemotherapy alone increased significantly from 9.7 to 28.1%(*P*<0.0001), especially for metastatic disease. The use of surgery alone for resectable disease increased over the study period, while the use of surgery plus radiotherapy did not change. Of the patients in this group, 73.0% had tumours resected: 64.1% with pTNM stage I or II, 25.2% stage IIIA and 8.6% stage IIIB or IV (2.1% unknown).

Chemotherapy used a single agent in 11.7% of the cases, two in 39.5%, three in 17.8% and four or more in 31.0%. Cisplatin was one of the drugs in 81.4% of the cases. The drugs used changed significantly over time: use of vindesine, cyclophosphamide and lomustine decreased significantly after 1986, while use of vinorelbine, ifosfamide and mitomycin-C increased significantly from 1986. The principal regimen during the first two periods was cisplatin-based chemotherapy with vindesine and/or cyclophosphamide and/or lomustine: 89.3% in 1982–85, and 64.7% in 1986–89. The most common regimen in the last two periods was the combination of cisplatin and vinorelbine: 29.2% in 1990–93, and 32.5% in 1994–97. Use of combined chemotherapy including cisplatin, ifosfamide and/or mitomycin-C began in 1990–93 and increased steeply over the last two periods: 8.4% in 1990–93, and 19.2% in 1994–97. Monotherapy with vinorelbine began in 1986–89, when it constituted 4.4% of the chemotherapy regimens, *vs* 15.0% in 1994–97.

### Simple regressive analysis of relative survival

Overall survival did not change significantly during the study period (*P*=0.861). Median survival time was 10.3 months during the first period and 10.2 months during the last. For resectable disease, however, improvement of survival over time was on the borderline of statistical significance (*P*=0.066): MST in this group increased from 21.1 months in 1982–85 to 35.8 months in 1990–93 and decreased slightly thereafter to 33.0 months in 1994–97. No differences from the first study period to the last were observed for locally advanced or metastatic disease (*P*=0.273; *P*=0.160), with MST 8.5 and 9.7 months, respectively, in locally advanced disease, and 4.6 and 4.4 months in metastatic disease.

Overall, the 1-, 2- and 5-year survival probabilities were, respectively, 45.9, 29.4 and 15.7%. For patients with resectable disease they were 71.3, 53.9 and 32.6%, and 79.2, 64.2 and 41.1% for those whose tumours were resected. The 1- and 2-year survival probabilities for all patients with locally advanced disease were 38.2 and 15.6%, for those treated by radiotherapy alone, 32.7 and 19.0%, and for those receiving radiotherapy and chemotherapy, 49.3 and 17.6%. These survival probabilities for all patients with metastatic disease were 16.8 and 5.2%, and for those treated with chemotherapy alone, 24.1 and 5.9%.

In all, 129 patients (7.5%) died within a month of diagnosis: 91 (70.5%) of them received no specific treatment.

Age (*P*<10^−5^), disease extent (*P*<10^−5^), histological subtype (*P*<10^−5^), and treatment (*P*<10^−5^) all had a significant influence on survival. Sex (*P*=0.840) and inclusion in a clinical trial (*P*=0.775) did not.

### Multiple regressive analysis of relative survival

The multiple regressive analysis considered age, sex, disease extent, histological subtype, period and treatment in a forward stepwise procedure.

We first included only the pretherapeutic patients' and disease's characteristics. Age over 70 years, advanced disease and mixed subtype were less favourable prognostic factors, and the adenocarcinoma and bronchioloalveolar carcinoma histological subtypes more favourable (Table 4).

Then we included, separately for each stage (Tables 5, 6, 7), characteristics of the patients, disease and treatment (because treatment depends strongly on stage). We excluded patients with bilateral disease or undetermined stage. We also excluded treatments administered to fewer than 20 patients, because there was not enough information to allow us to estimate the corresponding coefficient in the model (Table 3).

Age had no prognostic value in the models including treatment variables. Histology was a prognostic factor only in metastatic disease (Table 7): survival rate was higher for those with adenocarcinoma than with squamous-cell carcinoma. In resectable disease, survival was longer among patients undergoing only surgery or surgery and chemotherapy (Table 5). In locally advanced disease, the longest survival was observed with radiotherapy plus chemotherapy (Table 6).

In metastastic disease, patients treated with chemotherapy including new agents or classical agent with platin had a higher survival rate than patients treated by classical agent without platin or palliative care only (Table 7).

## DISCUSSION

The sex-ratio decreased significantly over time from 10.6/1 in 1982–85 to 6.7/1 in 1994–1997. Analysis of all French registries over this period shows the same trend in sex-ratio for lung cancer: 10.1/1 in 1980 and 6.0/1 in 1995 (Remontet *et al*, 2003). This trend illustrates the progressive burden of this disease in French women, related to their increasing regular use of tobacco since the end of the Sixties (Hill, 1998), more than two decades after women began smoking regularly in the US (US Public Health Service, 1980). The sex-ratio there is about 2/1 now, and lung cancer is the leading cause of cancer death in women (US Public Health Service, 2001).

The median age at diagnosis among the 1738 patients included in the sample was 63 years, lower than in other European population-based studies, which have found a median age of roughly 70 years (Gregor *et al*, 2001; Koyi *et al*, 2002; Mahmud *et al*, 2003). These series, however, unlike our study, included lung cancer patients (14–26%) not histologically confirmed, the confirmation rates declining with age (Koyi *et al*, 2002; Mahmud *et al*, 2003).

The distribution of disease extent also changed significantly during the study period. The percentage of patients with metastatic disease increased, as did the number of metastatic sites and the imaging procedures. This suggests that at least part of the increased frequency of metastatic disease may be due to stage migration, a well-known phenomenon (Feinstein *et al*, 1985). Cases classified as resectable accounted for 45.2% of the entire sample, and locally advanced stage for only 18.0%. This high percentage of resectable disease probably has two primary causes, the first being the absence of thorax CT scans during the early periods. Second, mediastinoscopy is not routine in France (Depierre *et al*, 2002), so that patients with stage IIIA disease often undergo surgery.

Disease management changed over the study period, especially for the locally advanced and metastatic stages. Surgery alone remained the primary treatment for resectable disease throughout the period. Combined treatments, such as surgery plus radiotherapy plus chemotherapy or surgery plus chemotherapy, remained relatively infrequent, as seen in a 10-year survey in the US (Fry *et al*, 1999). The meta-analysis published in 1995 (NSCLCCG, 1995) did not provide clear evidence that chemotherapy, especially adjuvant chemotherapy, is effective in prolonging survival in these cases. Similarly, only two small randomised studies of neoadjuvant chemotherapy were published during the study period (Rosell *et al*, 1994). Accordingly, no significant modification in the use of chemotherapy for resectable disease was to be expected.

Use of radiotherapy alone in resectable disease decreased during the study period, while surgery was increasingly used. This observation is consistent with other reports (Janssen-Heijnen and Coebergh, 2003). This trend may be related to advances in operative technique and perioperative care that have prompted greater recourse to surgery (Bolliger and Perruchoud, 1998). Postoperative radiotherapy in resected stage IIIAN2 disease is widely used in France as elsewhere (Emami and Perez, 1992) and did not change significantly during the study period.

The PORT meta-analysis confirmed the detrimental effect of postoperative radiotherapy in resected stage I and II disease and found its role in completely resected stage IIIA disease still unclear: an improvement of the local control but no effect on survival (PORT Meta-analysis Trialists Group, 1998).

The use of platinum-based chemotherapy in conjunction with radiotherapy in patients with locally advanced unresectable NSCLC has become standard in France since the publication by Le Chevalier *et al* (1991). In our study, chemotherapy plus radiotherapy in locally advanced disease increased significantly during the study period, from 21.2 to 59.1%. Inversely the use of radiotherapy alone decreased significantly, from 53.8 to 12.9%. Similar trends in the treatment of locally advanced NSCLC have been reported for the US (Fry *et al*, 1999).

The use of chemotherapy for metastatic disease also increased over time. Practices in our study were consistent with findings in the meta-analysis (NSCLCCG, 1995) with cisplatin-based chemotherapy being the most common first-line approach. Many studies reported that cisplatin-based combinations were associated with better response rates and sometimes better survival than monotherapy, and these combinations eventually became standard practice (Depierre *et al*, 1994; Le Chevalier *et al*, 1994). In our study, 11.7% of the patients treated by chemotherapy received one drug, 39.5% two, 17.8% three, and 31.0% four or more.

We did not see any improvement in overall survival between 1982 and 1997. In the retrospective analysis of patients with extensive NSCLC treated in the clinical trials of the SWOG between 1974 and 1988, an improved survival was observed for those treated with cisplatin-based chemotherapy (Albain *et al*, 1991), a treatment introduced during the 1980s. The European retrospective analysis of patients with advanced NSCLC enrolled in clinical trials between 1980 and 1991 observed no improvement in survival according to period of diagnosis (Paesmans *et al*, 1995). A study of 22 years of phase III trials for patients with advanced NSCLC (Breathnach *et al*, 2001) showed modest progress in survival for these patients: MST increased from 5.2 months in the early period (1973 to 1983) to 5.8 months in the later one (1984 to 1994).

Nevertheless, this improved survival from one clinical trial to another cannot be definitely attributed to improvement of therapies. The increasingly restrictive inclusion criteria may be a factor in the improved outcome of study subjects. These selection biases limit, of course, the generalisability of the results obtained in clinical trials to the real-life population of patients. Comorbidities, often linked to tobacco use, may prevent patients in the general population from receiving the optimal treatment (Janssen-Heijnen *et al*, 1998). The Will Rogers phenomenon (Feinstein *et al*, 1985) may also account for at least some of the improved survival observed in consecutive trials.

Lead time bias is another possible explanation for the improvements in survival observed in clinical trials. This bias is the only one of those mentioned here that is also possible in population-based studies, because the general population may also become more aware of lung cancer symptoms over time and consult earlier. Population-based studies are therefore of the utmost importance in assessing treatment efficiency and providing insight into public health policies.

Few population-based analyses of survival in patients with NSCLC have been published. MST of all patients with NSCLC in the SEER-database increased from 9.1 months in 1974–75 to 10 months in 1993–94 and the 3- and 5-year survival rates increased, respectively, by 2 and 4 points (Breathnach *et al*, 2001). A large hospital-based cancer registry showed no improvement in survival from 1985 through 1995 (Fry *et al*, 1999).

Elderly patients are excluded from clinical trials frequently and receive optimal treatment less often than younger patients (Earle *et al*, 2002). Their frequent comorbidities and frailty often preclude administration of the standard treatment (Janssen-Heijnen *et al*, 1998). But advanced age in itself does not contraindicate aggressive treatment (Deppermann, 2001; Langer *et al*, 2002). Age was not a prognostic factor in our study when we took into account the specific treatment administered. In various cancers, such as NSCLC (Foucher *et al*, 1993), studies using relative survival often dismiss age as a significant prognostic factor.

Performance status is probably the best single prognostic factor with stage in NSCLC (Albain *et al*, 1991). Unfortunately, in this population-based study there were too many missing values. It is not unlikely that more patients with bad PS were histologically diagnosed with lung cancer during the latter end rather than at the beginning of the study period as was demonstrated by UK cancer registries (Cartman *et al*, 2002). This could at least partially explain the absence of detection of an overall improvement in survival.

We did not observe any protective effect linked to female sex in our study. Several studies report better prognosis for women with NSCLC cancer than for men (Albain *et al*, 1991; Paesmans *et al*, 1995; Radzikowska *et al*, 2002), although not consistently (Keller *et al*, 2002).

Adenocarcinoma was associated with longer survival in the metastatic stage than squamous-cell carcinoma. The prognostic role of histological type remains controversial (Sculier *et al*, 1994; Charloux *et al*, 1997; Fry *et al*, 1999). Changes in pathology classifications over the years may account in part for these discrepancies.

Disease stage at diagnosis is the most important prognostic factor. Five-year relative survival for patients with resectable disease was 32.6%, regardless of the treatment. It was 41.1% for those who underwent surgery (alone or combined with other treatments), which can be compared to the 5-year crude survival in surgical series: 40.4% for the surgery arm, with or without postoperative radiotherapy in the IALT trial (The IALT Collaborative Group, 2004), and 43% in the surgery alone arm of the post-operative radiation therapy trial (Dautzenberg *et al*, 1999).

In our study, among patients with resectable disease, survival was shorter for those who could not undergo surgery and for those who had surgery and radiotherapy. This combined therapy, however, reflects their advanced pTNM stage: 64.6% of them had post-operative stage IIIA and 11.5% stage IIIB.

In locally advanced disease, the 1-year survival rate was 32.7% for patients treated by radiotherapy alone and 49.3% for those treated with radiotherapy plus chemotherapy. These results are similar to those previously reported in a clinical trial (Le Chevalier *et al*, 1991).

In metastatic disease, the 1-year survival rate was 16.8% for the overall sample, it was 24.1% for the patients who received only chemotherapy, a rate very close to that reported in the chemotherapy arms of clinical trials comparing chemotherapy to the best supportive care (NSCLCCG, 1995). Those patients with metastatic disease who were treated with platin-based chemotherapy had the best survival and in this group we observed a trend towards better results with the new agents combined with platin. The recently published phase III trials that included hundreds of patients with advanced NSCLC, did not demonstrate the superiority of any one combination of a platinum salt with a new agent over any other (Kelly *et al*, 2001; Schiller *et al*, 2002).

Overall, 16.7% of our patients received only palliative care. This percentage is to be compared to the 14% to 19% in the NCDB series (Fry *et al*, 1999), 43.2% in the Scottish population-based series (Gregor *et al*, 2001), and 39.8% in the Irish (Mahmud *et al*, 2003). The higher percentages in the two latter studies may be partly explained by the inclusion of patients with unconfirmed lung cancer and probably too frail to undergo diagnostic procedures.

Despite changes in treatments in accordance with the state of the art, survival did not improve. Nevertheless, the survival rates observed in our series are very similar to those reported in recent phase III clinical trials that include highly selected patients.

Several authors argue that SCLC is a more chemoresponsive disease than NSCLC, so that improved survival is more likely to be observed in patients with the former disease (Breathnach *et al*, 2001; Janssen-Heijnen and Coebergh, 2003). A previous study observed a significant improvement in survival in patients diagnosed with SCLC in the Bas-Rhin between 1981–83 and 1993–94 (Lebitasy *et al*, 2001).

These findings may also reflect the continuing pre-eminence of surgery in curing NSCLC. The benefits of chemotherapy in more advanced disease still appear too small to be translated into improved overall survival in an entire patient population, some being unable to receive chemotherapy. Thus, the impact of these newer treatments on survival will become apparent when a much greater proportion of patients will be treated with the appropriate treatment, and perhaps the final cohort was too early in the evolution of NSCLC management to outbalance the above factors.

## Figures and Tables

**Table 1:** Characteristics of the study sample

	**1982–85**		**1986–89**		**1990–93**		**1994–97**		
**Period**	**No.**	**%**	**No.**	**%**	**No.**	**%**	**No.**	**%**	***P***
*Sex* a									0.2044
Male	319	91.4	380	89.4	411	87.6	431	87.1	
Female	30	8.6	45	10.6	58	12.4	64	12.9	
									
*Age (years)*									0.0018
⩽55	82	23.5	101	23.8	93	19.8	108	21.8	
56–70	144	41.3	210	49.4	255	54.4	268	54.1	
>70	123	35.2	114	26.8	121	25.8	119	24.0	
									
Treatment on protocol^a^	11	3.2	6	1.4	20	4.3	36	7.3	0.0001
									
*Disease extent*									0.0267
Resectable	182	52.1	197	46.4	204	43.5	202	40.8	
Locally advanced	52	14.9	70	16.5	96	20.5	94	19.0	
Bilateral	7	2.0	13	3.1	6	1.3	12	2.4	
Metastastic	92	26.4	126	29.6	149	31.8	174	35.2	
Undetermined	16	4.6	19	4.5	14	3.0	13	2.6	
									
*Histology*									0.0003
Squamous-cell	229	65.6	282	66.4	300	64.0	267	53.9	
Adenocarcinoma	69	19.8	92	21.6	100	21.3	148	29.9	
Bronchioloalveolar	13	3.7	16	3.8	26	5.5	18	3.6	
Large-cell	35	10.0	34	8.0	41	8.7	51	10.3	
Mixed	3	0.9	1	0.2	2	0.4	11	2.2	

a Linear trend significant.

**Table 2:** Imaging procedures performed during the study period

	**1982–85**		**1986–89**		**1990–93**		**1994–97**		
**Period**	**No.**	**%**	**No.**	**%**	**No.**	**%**	**No.**	**%**	***P***
Fibrescopy	336	96.6	412	97.4	454	97.8	474	96.3	0.4999
Thoracic-CT scan^a^	72	20.7	177	42.0	402	86.8	472	96.3	<0.0001
Thoracic NMR^b^			10	2.4	12	2.6	11	2.3	0.9501
Abdominal US	321	92.8	376	90.4	425	92.6	425	89.3	0.1973
Adrenal CT scan^a^	18	5.2	83	20.0	220	48.1	245	51.6	<0.0001
Abdominal CT scan^a^	12	3.5	30	7.2	82	17.9	134	28.1	<0.0001
Brain CT^a^	33	9.6	160	38.5	313	67.7	386	80.9	<0.0001
Bone scan^a^	171	49.4	175	42.1	212	46.4	275	57.8	<0.0001
Brain scan^a^	67	19.4	16	3.9	0	0.0	0	0.0	<0.0001
Brain NMR^a^	1	0.3	3	0.7	7	1.5	10	2.1	0.0823
Mediastinoscopy	2	0.6	4	1.0	6	1.3	5	1.0	0.8181
Other	35	10.1	34	8.2	41	9.0	37	7.8	0.6643

a Linear trend significant.

b χ2 calculated from 1986. CT: computed tomography; US: ultrasound; NMR: nuclear magnetic resonance imaging.

**Table 3:** Therapeutic management over time

	**1982–85**		**1986–89**		**1990–93**		**1994–97**		
**Period**	**No.**	**%**	**No.**	**%**	**No.**	**%**	**No.**	**%**	***P***
*Resectable disease*									
Radiotherapy^a^	44	24.4	35	17.9	26	12.7	23	11.4	0.0023
Surgery^a^	60	33.3	99	50.5	108	52.9	112	55.4	<0.0001
Chemotherapy	2	1.1	2	1.0	5	2.5	6	3.0	0.4290
Surgery+radiotherapy	31	17.2	41	20.9	34	16.7	24	11.9	0.1149
Surgery+chemotherapy	11	6.1	5	2.6	6	2.9	8	4.0	0.2777
Chemotherapy+radiotherapy	13	7.2	1	0.5	2	1.0	8	4.0	0.0004
Surgery+radio+chemotherapy	8	4.4	3	1.5	11	5.4	10	5.0	0.2043
Supportive care	11	6.1	10	5.1	12	5.9	11	5.4	0.9745
									
*Locally advanced disease*									
Radiotherapy^a^	28	53.8	49	70.0	39	40.6	12	12.9	<0.0001
Chemotherapy	7	13.5	3	4.3	12	12.5	11	11.8	0.2720
Chemotherapy+surgery	0	0.0	0	0.0	0	0.0	2	2.2	0.2419
Chemo+radiotherapy^a^	11	21.2	9	12.9	29	30.2	55	59.1	<0.0001
Chemo+surgery+radiotherapy	0	0.0	3	4.3	6	6.3	4	4.3	0.3247
Supportive care	6	11.5	6	8.6	10	10.4	9	9.7	0.9552
									
*Metastatic disease*									
Radiotherapy^a^	28	31.1	30	24.6	17	11.5	8	4.6	<0.0001
Chemotherapy^a^	24	26.7	32	26.2	64	43.2	113	65.3	<0.0001
Chemotherapy+surgery	0	0.0	1	0.8	1	0.7	2	1.2	0.9155
Chemo+radiotherapy^a^	17	18.9	4	3.3	15	10.1	10	5.8	0.0003
Surgery	0	0.0	0	0.0	1	0.7	0	0.0	0.6754
Chemo+radiotherapy+surgery	0	0.0	0	0.0	0	0.0	1	0.6	0.9999
Supportive care	21	23.3	55	45.1	50	33.8	39	22.5	0.0002

a Linear trend significant

**Table 4:** Multiple regressive survival analysis for pretherapeutic characteristics

**Variables**	**Hazard ratio**	**CI**	***P***
*Sex*			0.47526
Male	1		
Female	0.938	0.786; 1.120	
			
*Age (years)*			0.00005
⩽55	1		
56–70	1.047	0.914; 1.199	
>70	1.376	1.177; 1.610	
			
*Histology*			0.00243
Squamous-cell	1		
Adenocarcinoma	0.844	0.733; 0.971	
Bronchioloalveolar	0.645	0.469; 0.886	
Large-cell	0.972	0.807; 1.171	
Mixed	1.758	1.022; 3.026	
			
*Disease extent*			<10^−5^
Resectable disease	1		
Locally advanced disease	2.785	2.386; 3.251	
Metastatic disease	5.410	4.706; 6.219	
Bilateral disease	2.079	1.451; 2.978	
Undertermined	6.125	4.559; 8.229	
			
*Period*			0.46030
1982–85	1		
1986–89	0.978	0.831; 1.152	
1990–93	0.897	0.764; 1.054	
1994–97	0.911	0.777; 1.068	

**Table 5:** Multiple regressive survival analysis for pretherapeutic and therapeutic variables in resectable disease

**Variables**	**Hazard ratio**	**IC**	***P***
*Sex*			0.21884
Male	1		
Female	0.811	0.575; 1.142	
			
*Age (years)*			0.79369
⩽55	1		
56–70	1.025	0.802; 1.310	
>70	0.936	0.679; 1.291	
			
*Histology*			0.88776
Squamous-cell	1		
Adenocarcinoma	0.952	0.741; 1.221	
Bronchioloalveolar	0.913	0.576; 1.449	
Large-cell	1.069	0.718; 1.591	
Mixed	1.481	0.608; 3.605	
			
*Treatments*			<10^−5^
Surgery alone	1		
Surgery+radio+chemotherapy	1.656	1.055; 2.598	
Chemo+radiotherapy	3.761	2.336; 6.055	
Surgery+chemotherapy	0.888	0.497; 1.589	
Surgery+radiotherapy	1.944	1.492; 2.533	
Radiotherapy alone	3.534	2.640; 4.730	
Supportive care alone	5.353	3.637; 7.878	
			
*Period*			0.49530
1982–85	1		
1986–89	1.013	0.778; 1.319	
1990–93	0.837	0.635; 1.104	
1994–97	0.949	0.724; 1.245	

**Table 6:** Multiple regressive survival analysis for pretherapeutic and therapeutic variables in locally advanced disease

**Variables**	**Hazard ratio**	**IC**	***P***
*Sex*			0.07994
Male	1		
Female	1.538	0.972; 2.435	
			
*Age (years)*			0.60239
⩽55	1		
56–70	1.155	0.845; 1.579	
>70	1.035	0.705; 1.519	
			
*Histology*			0.68538
Squamous-cell	1		
Adenocarcinoma	1.147	0.802; 1.641	
Large-cell	0.975	0.635; 1.498	
Mixed	2.301	0.516; 10.267	
			
*Treatment*			0.00009
Chemo+radiotherapy	1		
Chemotherapy alone	2.227	1.448; 3.426	
Radiotherapy alone	1.457	1.034; 2.053	
Supportive care alone	3.560	2.238; 5.665	
			
*Period*			0.59819
1982–85	1		
1986–89	0.796	0.536; 1.183	
1990–93	0.974	0.678; 1.399	
1994–97	1.002	0.675; 1.487	

**Table 7:** Multiple regressive survival analysis for pretherapeutic and therapeutic variables in metastatic disease

**Variables**	**Hazard ratio**	**IC**	***P***
*Sex*			0.38535
Male	1		
Female	0.895	0.696; 1.152	
			
*Age (years)*			0.63040
⩽55	1		
56–70	0.908	0.725; 1.136	
>70	0.979	0.745; 1.287	
			
*Histology*			0.01699
Squamous-cell	1		
Adenocarcinoma	0.751	0.607; 0.929	
Bronchioloalveolar	0.586	0.351; 0.978	
Large-cell	1.023	0.788; 1.330	
Mixed	1.361	0.553; 3.347	
			
*Treatments*			<10^−5^
Chemotherapy alone with classical agents and platin	1		
Chemotherapy alone with recent agents and platin	0.749	0.547; 1.026	
Chemotherapy alone with recent agents w/o platin	1.069	0.722; 1.583	
Chemotherapy alone with classical agents w/o platin	2.010	1.142; 3.540	
Chemo+radiotherapy	0.727	0.501; 1.055	
Radiotherapy alone	1.359	0.982; 1.881	
Supportive care alone	2.316	1.749; 3.067	
			
*Period*			0.23791
1982–85	1		
1986–89	0.981	0.732; 1.315	
1990–93	1.088	0.821; 1.442	
1994–97	1.272	0.949; 1.704	
